# Multi-Energy Load Prediction Method for Integrated Energy System Based on Fennec Fox Optimization Algorithm and Hybrid Kernel Extreme Learning Machine

**DOI:** 10.3390/e26080699

**Published:** 2024-08-17

**Authors:** Yang Shen, Deyi Li, Wenbo Wang

**Affiliations:** 1 College of Science, Wuhan University of Science and Technology, Wuhan 430081, China; shenyang@wust.edu.cn; 2 Hubei Province Key Laboratory of System Science in Metallurgical Process, Wuhan University of Science and Technology, Wuhan 430065, China; lideyi202312@163.com

**Keywords:** multi-energy load prediction, integrated energy system, comprehensive weight method, fennec fox optimization algorithm, hybrid kernel extreme learning machine

## Abstract

To meet the challenges of energy sustainability, the integrated energy system (IES) has become a key component in promoting the development of innovative energy systems. Accurate and reliable multivariate load prediction is a prerequisite for IES optimal scheduling and steady running, but the uncertainty of load fluctuation and many influencing factors increase the difficulty of forecasting. Therefore, this article puts forward a multi-energy load prediction approach of the IES, which combines the fennec fox optimization algorithm (FFA) and hybrid kernel extreme learning machine. Firstly, the comprehensive weight method is used to combine the entropy weight method and Pearson correlation coefficient, fully considering the information content and correlation, selecting the key factors affecting the prediction, and ensuring that the input features can effectively modify the prediction results. Secondly, the coupling relationship between the multi-energy load is learned and predicted using the hybrid kernel extreme learning machine. At the same time, the FFA is used for parameter optimization, which reduces the randomness of parameter setting. Finally, the approach is utilized for the measured data at Arizona State University to verify its effectiveness in multi-energy load forecasting. The results indicate that the mean absolute error (MAE) of the proposed method is 0.0959, 0.3103 and 0.0443, respectively. The root mean square error (RMSE) is 0.1378, 0.3848 and 0.0578, respectively. The weighted mean absolute percentage error (WMAPE) is only 1.915%. Compared to other models, this model has a higher accuracy, with the maximum reductions on MAE, RMSE and WMAPE of 0.3833, 0.491 and 2.8138%, respectively.

## 1. Introduction

Energy is a key factor in promoting economic growth, providing the foundation for the development of human society, and plays an extremely important role in human society [1]. However, fossil energy sources are facing a growing shortage. At the same time, the problems of inefficiency, energy waste and environmental pollution of traditional thermal power generation are becoming more and more prominent. Therefore, there is an urgent need for the energy system to accelerate its transformation [2]. Traditional energy system construction has largely been an extension in depth within single systems. There is minimal physical interconnection and information interaction between these systems, which hinders the complementary and mutually advantageous use of diverse energy sources. In order to cope with these shortcomings and promote the development of renewable energy sources, the integrated energy system is born at the right moment [3,4].

The IES reduces the use of conventional energy sources and increases the proportion of renewable energy sources to meet the targets of carbon peaking and carbon neutrality [5]. It has an efficient and flexible energy supply mode, coupling various modalities of energy, for instance, electricity, cooling and heating loads. Through the interconnection and optimized scheduling of energy, the efficient use and sustainable development of energy in the whole system are greatly realized [6]. In this intelligent energy system, accurate short-term forecasting of the user-side multi-energy load is extremely important for maintaining the balance between energy supply and demand, optimizing energy dispatch, and ensuring the safe and stable operation of the system [7]. This need has prompted it to become a significant area in current academic research. 

It has an important practical value to carry on an accurate multi-energy load prediction. In recent years, many scholars have focused on tackling the issues of accuracy and reliability in IES multi-energy load prediction, proposing numerous load predictive methodologies. As a result, the field has seen a gradual enrichment of research outcomes. Current load forecasting methods mainly include two categories [8,9,10]. The former are traditional linear prediction methods, such as the exponential smoothing method [11], linear regression method [12], time series method [13], Kalman filter algorithm [14], etc. Such methods often fail to effectively explore the inherent characteristics of power load data, and it is difficult to achieve the increasing accuracy of load prediction. The latter are advanced machine learning ways, for instance, fuzzy theory [15], support vector machine [16], artificial neural network [17,18,19], deep neural network [20,21,22,23], etc. These methods are highly capable and adaptive to learn the intrinsic correlation between data efficiently and have been widely used in the domain of IES multi-energy load prediction. In reference [24], the convolutional neural network (CNN) is first applied to fetch hidden features, and the features are taken as inputs. A bidirectional gated recurrent unit (BIGRU) network is used to forecast IES electricity, cooling and heating loads, and three attention mechanism modules are combined to enhance the impact generated by the important information. In reference [25], a multi-task learning architecture with homoscedasticity uncertainty and a multiple load prediction model integrating multiple gated cyclic unit neural network (GRU) strategies was constructed, which could realize the sharing of different network results according to the volatility degree in different loads. Nevertheless, the forecasting accuracy is not high due to the failure to consider weather and other related factors. Meteorological factors, as important input features for load forecasting, to some extent affect users’ energy demand, which in turn affects load power. In reference [26], correlation analysis between multiple loads and between multiple loads and meteorological elements is carried out and then a long short-term memory network (LSTM) is applied to subsequent forecasting work. However, deep neural networks increase the complexity and difficulty of the training process. There are many hyperparameters to be tuned [27], which leads to a high training time and computational cost when processing large-scale data, and takes a long time to converge and achieve the best performance. The extreme learning machine (ELM) is trained in the manner of random initialization without complex iterative optimization process, and the training speed is very fast [28,29]. Moreover, there is no need to adjust a quantity of hyperparameters, simplifying the design and realization for the model. The kernel extreme learning machine is an improvement based on ELM, which introduces kernel function mapping to replace the random mapping relationship between the input and output of the hidden layer. While retaining the good predictive performance of ELM, it avoids the problem of large randomness in training results caused by manually setting the number of nodes, and has better robustness [30]. However, a single kernel function has the disadvantage that it is hard to completely adjust to various data features. In references [31,32,33], a hybrid kernel extreme learning machine (HKELM) was obtained by combining the advantages of different kernel functions and weighting. It has the performance of efficiently dealing with intricate nonlinear characteristics, and can readily strengthen the final forecasting effect. However, the HKELM model is highly sensitive to the selection of kernel parameters and penalty coefficients [34,35,36]. The difficulty of visually identifying the best parameter combinations may lead to a loss of accuracy in the prediction results.

Aiming at the problems of numerous meteorological factors that affect load forecasting and the strong randomness of manually selecting HKELM kernel parameters and penalty coefficients, this paper proposes an IES multi-energy load forecasting method using the FFA-HKELM model. Firstly, the comprehensive weight method integrates the entropy weight method with Pearson correlation coefficients to evaluate the relevance and information content of the features concurrently. This approach reduces the dependence on a single feature selection criterion. The influential factors that are highly related to the three types of loads are selected as model features and input into the HKELM weighted by two kernel functions for training. Then, the key parameters for the model are optimized employing a novel FFA method to obtain the FFA-HKELM model with optimal parameter combinations. Eventually, the model is adopted in the actual integrated energy system of the Tempe area in the United States, and the electrical, heating and cooling loads are forecast in the short term. Comparing the results with other forecasting methods, it is verified that this method can better realize the accurate prediction of future multiple load values through the analysis of multiple evaluation indexes. 

This article is arranged as follows. Section 2 describes the theory of the combined weights approach and the analysis of the relevant factors in the IES. Section 3 introduces the FFA algorithm, HKELM and the process of establishing the FFA-HKELM prediction model. Meanwhile, the optimization effect of the FFA algorithm is compared with other algorithms. Section 4 analyzes the predictive performance of this model and other comparative models. Section 5 is the summary of this paper and the future research plan.

## 2. Analysis of IES Structure and Feature Variable Screening

### 2.1. IES Structure

**Figure 1:**
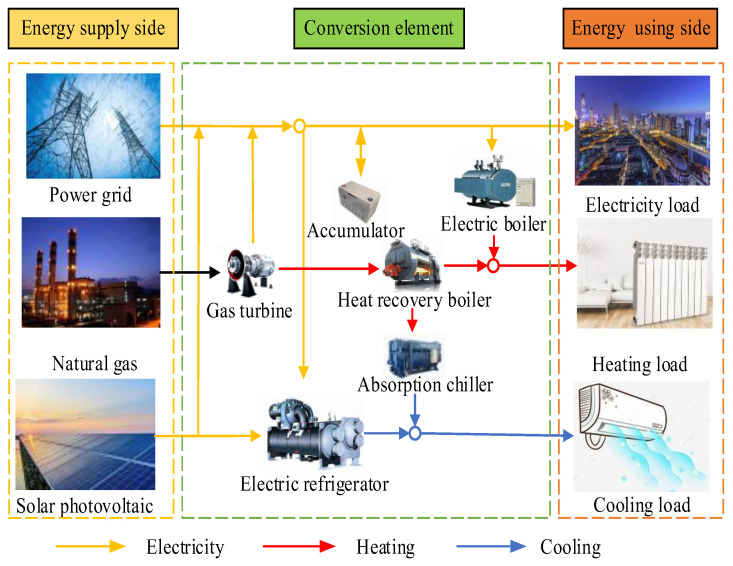
Schematic diagram of IES structure.

IES, as a new type of energy system, optimizes the whole process of energy production, transmission, storage and use. It can efficiently harness renewable energy sources, ensuring their optimal use. Additionally, it achieves gradient utilization of non-renewable energy sources, promoting the synergistic operation of various energy types. This approach enhances the overall energy utilization efficiency [37]. The schematic diagram of the system is shown in Figure 1. The electrical loads are provided by gas turbines, storage batteries, photovoltaic devices and external grids. The cooling load is provided by electric chillers and absorption chillers. The heating load is mainly provided by electric boilers and waste heat boilers.

**Figure 2:**
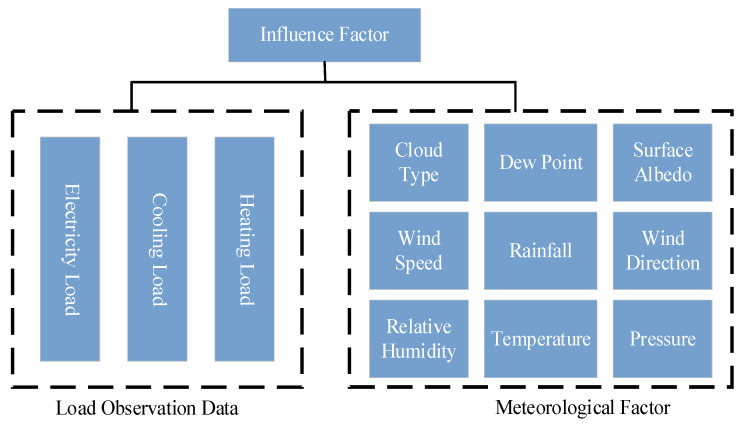
Multi-energy load influencing factors.

During the energy conversion process, the consumption by various types of loads necessitates the collaborative functioning of multiple energy subsystems. This means that different energy subsystems interact with each other. Therefore, there is a certain degree of close relationship between electrical, heating and cooling loads in the IES. In addition, terminal users have uncertainties when using different loads, which are readily impacted by meteorological elements, for instance, rainfall and temperature. Climate change directly affects user demand for workload. When selecting input characteristics, external meteorological factors should be taken into account, all of which are shown in Figure 2.

### 2.2. Entropy Weight Method

Entropy weighting is an objective weighting method, which can fully explore the inherent laws and information of the original data, and the final weights will not be affected by the subjective experience or preference of the decision maker.

In information theory, entropy is a measure of uncertainty, and the degree of randomness and disorder can be determined by calculating the entropy value. A smaller information entropy value for an indicator signifies a higher degree of variability, implying that it offers more information. Consequently, this indicator assumes a more significant role in comprehensive evaluation, which is reflected in its greater weight. On the contrary, the greater the information entropy of an indicator, the lower the variability of the indicator and the less information it provides. Therefore, the smaller the role it can play in the comprehensive evaluation, the lower the corresponding weight. The detailed calculation process is as follows:

- Construct a standardized matrix of indicator data: The index data of the initial data matrix are normalized.
- Computational information entropy: The output entropy of each index is calculated from the normalized standard matrix. The entropy value can be calculated as follows:
  (1) $$e=-\frac{1}{\ln m}\sum d_{j}\ln d_{j}$$
  where e represents the information entropy of the index; m indicates the number of all indicators; $d_{j}$ represents the normalized value of the indicator.
- Determine index weight: The information entropy of each index is used to determine its weight. The weight is calculated as follows:
  (2) $$\omega_{j}=\frac{1-e_{j}}{\sum_{j=1}^{m}\left(1-e_{j}\right)}$$
  where $\omega_{j}$ represents the weight of an indicator.

The information weight is a key measure of the information content of an indicator. A high weight means that the indicator has a significant impact on the decision-making outcome. Using the calculation of information entropy and weights, it is possible to set a weight distribution for decision-making tasks. This helps decision makers to make informed decisions among different indicators and enhances the accuracy and reliability of decision making. However, information weighting mainly focuses on the diversity and uncertainty of indicators without fully considering the interactions between them. Therefore, in practical applications, it is essential to fully consider the combined impact of indicators in conjunction with other assessment methods and expertise.

As can be seen from Table 1, the weight value of Cloud Type is as high as 0.423, indicating that the dispersion of data distribution of this indicator is enhanced, and thus contains more abundant information. The weights of Cooling, Heating, Surface Albedo, Relative Humidity, Rain, Wind Speed, Wind Direction, Temperature and Electricity are 0.1017, 0.0746, 0.0740, 0.0740, 0.0684, 0.0528, 0.0401, 0.0313 and 0.0278, respectively. The weights of Dew Point and Pressure are 0.0165 and 0.0158, respectively, indicating a low degree of variability in these two indicators.

### 2.3. Pearson Correlation Coefficient Method

However, considering the comprehensiveness of the influencing factors and using all elements as potential input features will bring too much noise during the feature selection process for IES multi-energy load forecasting. It leads to some extraneous elements being added to the model, which often brings negative impacts and instead reduces the computational efficiency and prediction accuracy [38]. Therefore, so as to quantitatively analyze the influence level with internal and external factors and efficiently screen out the key influence indexes, this paper adopts the Pearson correlation coefficient to analyze the degree of relevance between electricity, cooling, heating loads and external meteorological factors. Assuming that there are two sample sequences $X_{i}$ and $Y_{i}$, the specific expression of this coefficient is as follows:

(3) $$P_{XY}=\frac{\sum_{i=1}^{M}\left(X_{i}-\bar{X})(Y_{i}-\bar{Y}\right)}{\sqrt{\sum_{i=1}^{M}{\left(X_{i}-\bar{X}\right)}^{2}}\sqrt{\sum_{i=1}^{M}{\left(Y_{i}-\bar{Y}\right)}^{2}}}$$

where M is the amount of data for each group of samples. $\bar{X}$ and $\bar{Y}$ refer to the mean value of sequences $X_{i}$, $Y_{i}$, severally. The value of $P_{XY}$ ranges from −1 to 1.

The Pearson correlation coefficients between the electrical, heating, cooling loads and the meteorological data of the IES in the Tempe area are obtained by calculations and the consequences of the correlations are presented in Table 2.

As shown in Table 2, the correlation between electrical and heating loads of the IES in the Tempe area is extremely strong. The strong degree of relevance between heating and cooling loads as well as between heating and electrical loads indicates that any two loads interact with each other. At the same time, there is a strong degree of relevance between electric, heating, cooling loads and dew point, rainfall, temperature and pressure, and a weak correlation with cloud type, surface albedo, wind direction, humidity and wind speed. Among them, the absolute value of the obtained Pearson correlation coefficients for temperature and precipitation, as key indicators directly related to the production and life of the users, is more than 0.55, illustrating that the impact of these two factors on the changes in load is particularly prominent. The positive correlation between pressure and cooling load is high, indicating that pressure positively affects users’ cooling habits. 

### 2.4. Comprehensive Weight Method Analysis of Multi-Energy Load Relevant Factors

The composite weight method is a variable screening technique that incorporates the entropy weight method and the Pearson correlation coefficient. This method is designed to assess and select characteristic variables by integrating the Pearson correlation coefficient with the information weight of each variable. It determines their combined weights through this integrated evaluation, providing a comprehensive measure of their significance. The Pearson correlation coefficient of each characteristic variable is multiplied with its entropy weight to arrive at its composite weight value. The calculation of the composite weights is based on the following formula:

(4) $$F_{EC}=F_{EC}\times F_{PCC}$$

where $F_{EC}$ represents comprehensive weight, $F_{EC}$ represents information weight, and $F_{PCC}$ represents the Pearson correlation coefficient. The higher the $F_{EC}$ value of the variable, the more critical the role it plays in predicting the target variable, and the stronger the correlation.

**Figure 3:**
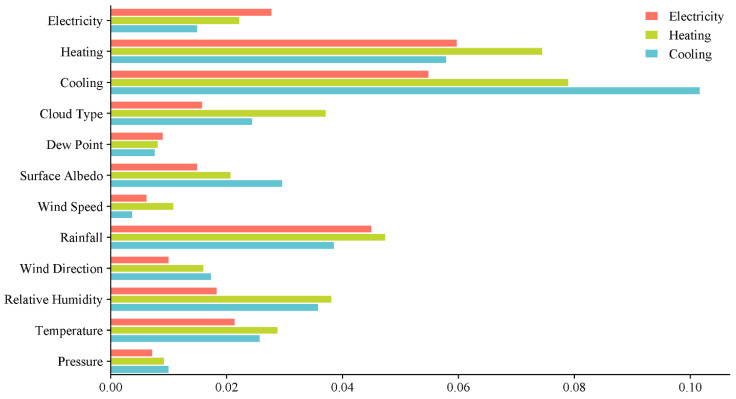
The comprehensive weight of each characteristic variable.

The advantage of the comprehensive weighting method is that it not only covers the consideration of linear relationships, but also includes the analysis of key factors, providing a more comprehensive evaluation and screening framework for characteristic variables. This method enhances the accuracy and reliability of feature selection. Based on the results of the combined weight calculations presented in Table 3 and Figure 3, the following input variables were selected to predict the different target variables:

- Electricity prediction: Electricity, Heating, Cooling, Cloud Type, Surface Albedo, Rainfall, Wind Direction, Relative Humidity, Temperature;
- Heating prediction: Electricity, Heating, Cooling, Cloud Type, Surface Albedo, Wind Speed, Rainfall, Wind Direction, Relative Humidity, Temperature;
- Cooling prediction: Electricity, Heating, Cooling, Cloud Type, Surface Albedo, Rainfall, Wind Direction, Relative Humidity, Temperature, Pressure.

These input variables are selected based on their combined weights, taking into account the Pearson correlation coefficient and information weights. The integration of these variables into the forecasting model is expected to increase the accuracy and stability of electrical, heating and cooling loads forecasting.

## 3. Multi-Energy Load Prediction Model Using FFA-HKELM

### 3.1. Fennec Fox Optimization Algorithm

The fennec fox optimization algorithm is an innovative heuristic algorithm suggested by Trojovsky [39] in 2022, which simulates the predation behavior of fennec foxes in nature for calculation. Comparing the effects with the conventional optimization algorithm, it can achieve convergence at a faster rate and find a better plan in a shorter time. And in high-dimensional spaces, it can effectively avoid a slide into local optimal solutions. The key strategy for the FFA algorithm is divided into two phases: development and exploration, specifically, the natural behavior of the fennec foxes in digging up the sand in search of prey and avoiding predator attacks.

Firstly, the fennec fox population is randomly initialized according to the boundary of this question, and the concrete procedure is the following:

(5) $$\left\{ \begin{array}{l} x_{i,j}=lb_{j}+r\cdot (ub_{j}-lb_{j})\\ i=1,2,\cdots ,M;j=1,2,\cdots ,N \end{array}\right.$$

where $x_{i,j}$ refers to the control variable. M refers to the number of all fennec foxes. N is the quantity of control variables. The interval of random digit r is [0, 1]. $lb_{j}$ and $ub_{j}$ refer to the upper and lower boundaries of the control variable, severally.

After initializing the population, the individual position of the population is updated in the search space according to the two stages of the fennec fox’s natural behavior. The specific process is as follows:

**The first stage is development**. At night, fennec foxes hunt alone, using their large ears to detect potential prey under the sand. Once they find their target, they burrow with their feet, leaving prey exposed for easy capture. This hunting behavior can be regarded as a local search, and simulating this behavior can help to improve the problem-solving ability and thus increase the possibility of obtaining a globally optimal solution. Assume a neighborhood of radius R centered on the actual position of the fennec fox. The fennec fox searches locally in this area to obtain the optimal solution, and its position update formula is shown below.

(6) $$\{\begin{array}{c} x_{i,j}^{P_{1}}=x_{i,j}+R_{i,j}\cdot (2\cdot r-1)\\ R_{i,j}=\alpha \cdot x_{i,j}\cdot (1-\frac{t}{T})\;\; \end{array}$$

where $x_{i,j}^{P_{1}}$ refers to the new location of the fennec fox after updating for the development phase. t refers to the present iteration amount. α is the constant 0.2. T refers to the maximum iteration amount. $R_{i,j}$ refers to the radius of the neighborhood of $x_{i,j}$.

If the objective function deserves to be improved, then the location of the fennec fox will also change. The concrete procedure is the following:

(7) $$X_{i}=\left\{ \begin{array}{l} X_{i},F_{i}\leq F_{i}^{P_{1}}\\ X_{i}^{P_{1}},F_{i}^{P_{1}}<F_{i} \end{array}\right.$$

where $F_{i}^{P_{1}}$ refers to the outcome of the objective function in this development phase. $X_{i}^{P_{1}}$ refers to the new position of the fennec fox in the first phase.

The second stage is exploration. Fennec foxes may be prey for feral predators, for instance, caracals and hyenas. Despite this, they are able to successfully evade these predators with their incredible speed of movement. This evasion strategy becomes the basis of the global search, and simulating this strategy helps to avert a slide into local optimal solutions. Then, for the second stage, the locations of fennec foxes are updated according to Formulas (8) and (9).

(8) $$x_{i,j}^{rand}=x_{k,j},k\in \left\{1,2,\ldots ,M\right\},i=1,2,\ldots ,M$$

(9) $$x_{i,j}^{P2}=\left\{ \begin{array}{l} x_{i,j}+r\cdot (x_{i,j}-{x_{i,j}}^{rand}),F_{i}\leq F_{i}^{rand}\\ x_{i,j}+r\cdot ({x_{i,j}}^{rand}-I\cdot x_{i,j}),F_{i}^{rand}<F_{i} \end{array}\right.$$

where $x_{i,j}^{rand}$ refers to the location of the fennec fox’s escape target. $x_{i,j}^{P2}$ refers to the fennec fox after the update of the exploration phase. $F_{i}^{rand}$ refers to the outcome of the objective function. The interval of the random number I is [1,2].

If the objective function value becomes better at this location, the process of updating the location of the fennec fox is the following:

(10) $$X_{i}=\left\{ \begin{array}{l} X_{i}^{P2},F_{i}^{P2}<F_{i}\\ X_{i},F_{i}\leq F_{i}^{P2} \end{array}\right.$$

where $X_{i}^{P2}$ is the new position of the fennec fox in the second stage. $F_{i}^{P_{2}}$ is the value of the objective function in the exploration stage.

### 3.2. FFA Performance Evaluation

The pseudo-code of the specific implementation steps of the FFA algorithm is in Algorithm 1. So as to investigate the effectiveness of the FFA algorithm, this article compares the optimization results of this algorithm with the grey wolf optimizer algorithm (GWO) [40], whale optimization algorithm (WOA) [41] and sparrow search algorithm (SSA) [42], severally. The download address is https://seyedalimirjalili.com/projects (accessed on 15 January 2024). The quantity of populations and the maximum quantity of iterations for whole algorithms are considered as 30 and 500. The experiments are performed in MATLAB R2021a software using a 64-bit Core i5 processor with 2.50 GHz and 16 GB main memory in Windows 11.
**Algorithm 1** Pseudo-code of the FFA algorithm**Start FFA**.1.Input the details of the optimization problem.2.Establish the parameters by defining the total number of iterations (T) and the size of the fennec fox population (N).3.Initialize the positions of the fennec foxes and commence the assessment of the objective function.4.For t=1:T.5.For i=1:N.**6**.**Stage 1: Dig through the sand to find prey (development)**.7.Calculate the new state of the *i*th fennec fox using Formula (6).8.Update the *i*th fennec fox using Formula (7).**9**.**Stage 2: Face predator attacks using escape strategies (exploitation)**.10.Generate the target location for the *i*th fennec fox to escape and evaluate its objective function using Formula (8).11.Calculate the new state of the *i*th fennec fox using Formula (9).12.Update the *i*th fennec fox using (10).13.end for i=1:N14.Save the best candidate solution so far.15.end for t=1:T16.Output the optimal solution of the given optimization problem.**End FFA.**

**Figure 4:**
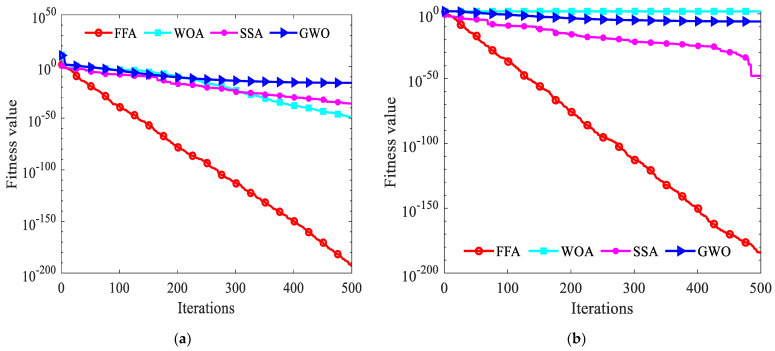

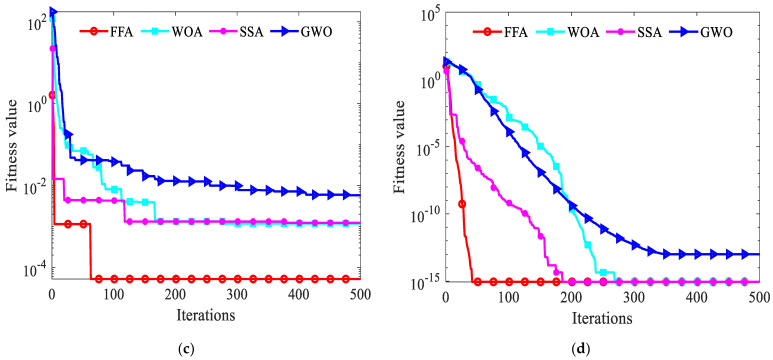
(**a**) The first benchmarking function; (**b**) the second benchmarking function; (**c**) the third benchmarking function; (**d**) the fourth benchmarking function.

Different benchmarking functions are also selected so as to comprehensively verify the reliability of the optimization algorithms. Four distinct algorithms are independently executed 30 times each. The average of these results is taken as the final outcome. This approach effectively negates the influence of random factors, thereby enhancing the credibility and persuasiveness of the experimental findings. The convergence curves of all algorithms are illustrated in Figure 4.

### 3.3. HKELM Algorithm

**Figure 5:**
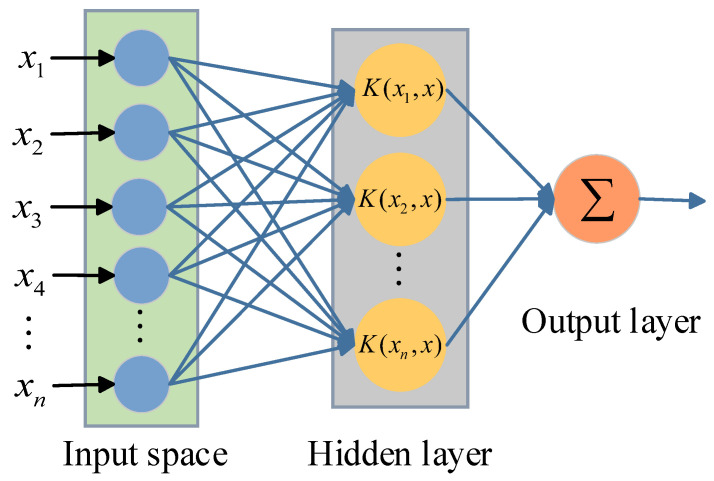
KELM network structure diagram.

ELM is a feedforward neural network based on a single layer that creates weights and thresholds at random. During the training process, a unique and optimal solution can be obtained by adjusting only the number of hidden layer neurons without making any other adjustments. Therefore, ELM has better convergence speed and efficiency. However, when there are multiple unknown datasets in the model, the accuracy of ELM is relatively low. The kernel extreme learning machine (KELM) is an improved algorithm developed by combining ELM and kernel function. It not only fully retains the original advantages of ELM, but also improves the prediction performance and stability of the model. Its network framework is as illustrated in Figure 5, where K is the kernel function matrix.

The expression for the KELM output model is shown below.

(11) $$f(x)=h(x)\beta =h(x)H^{T}{\left(\frac{I}{C}+HH^{T}\right)}^{-1}T$$

where f(x) is network output. h(x) and H are mapping matrices. C refers to the penalty coefficient. T refers to the aim vector. I refers to the unit matrix.

The kernel function in this model can be defined by the following Equation (12).

(12) $$\{\begin{array}{c} \Omega =HH^{T}\;\;\;\;\;\;\;\\ \Omega_{i,j}=h(x_{i})h(x_{j})=K(x_{i},x_{j}) \end{array}$$

The input data are transformed into feature vectors in a high-dimensional space by mapping the kernel function, which is thus obtained:

(13) $$f(x)=\left[\begin{array}{c} K(x,x_{1})\\ \vdots \\ K(x,x_{n}) \end{array}\right]{\left(\Omega +\frac{I}{C}\right)}^{-1}T$$

Radial basis kernel function and polynomial kernel function are two common kernel functions; the specific expressions are shown below.

(14) $$\{\begin{array}{c} K_{Poly}(x,x_{i})={\left(m(x\cdot x_{i})+n\right)}^{p}\\ K_{RBF}(x,x_{i})=\exp(-\frac{{\left\|x-x_{i}\right\|}^{2}}{2\sigma^{2}}) \end{array}$$

where m,n and p are the kernel parameters of the polynomial kernel function, respectively. σ is the nuclear parameter of the radial basis kernel function.

The selection of appropriate kernel functions has a significant impact on the performance of the KELM model; therefore, finding a suitable kernel function is crucial for the KELM model. So as to comprehensively heighten the generalization and learning ability of KELM, this article combines the radial basis kernel function and polynomial kernel function as the kernel function of the KELM model by linear combination, and combines the benefits of these two kernel functions to obtain a new HKELM model, whose expression is as follows:

(15) $$\left\{ \begin{array}{l} K_{hybrid}(x,x_{i})=\omega K_{Poly}(x,x_{i})+(1-\omega )K_{RBF}(x,x_{i})\\ \omega \in [0,1] \end{array}\right.$$

where ω is the weight coefficient of HKELM.

### 3.4. Establishment of FFA-HKELM Prediction Model

**Figure 6:**
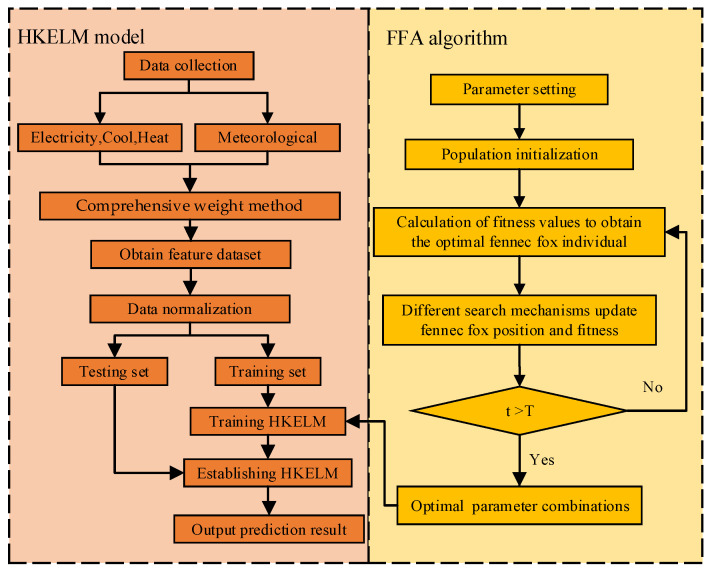
Frame structure diagram of FFA-HKELM model.

The precision of prediction for the HKELM model is determined by the mixed kernel parameters ω,m,n,σ and penalty parameters C, so it is particularly important to optimize these key parameters. Here, the FFA method is used for optimization, and the FFA-HKELM model is established to achieve the prediction of the electric cooling and heating loads. The whole framework procedure is as illustrated in Figure 6, and the specific process is the following.

(1) The comprehensive weighting method is applied to combine the entropy weight method and Pearson correlation coefficient, fully considering the correlation and information of each feature, in order to select appropriate input features to build a multivariate time series for IES load forecasting.

(2) The dataset is divided and normalized, and the resulting training set is input into the FFA-HKELM model to seek out the optimal parameter combinations by means of the FFA algorithm.

(3) Set the parameters of the FFA algorithm, namely, population quantity N, the quantity of iterations T, variable dimension d, and upper and lower limits [lb,ub].

(4) Initialize the population by combining all the mixed core parameters and punishment parameters as the position of each fennec fox, and initialize the position of individual fennec foxes by Formula (5).

(5) Calculate and sort the fitness values of individual fennec foxes, find out the lowest fitness value as the optimal fennec fox individual, and the mean square error is considered as the fitness function.

(6) Update the location and fitness of the fennec fox population. The numerical value of fitness for the current iteration optimal fennec fox is in comparison with the value of the last iteration optimal fennec fox. If the result is better than the previous generation, it is updated; otherwise, it is unchanged.

(7) Determine if the quantity of iterations has been reached. In case the quantity is reached, the process ends. Input the optimal mixed kernel parameters and penalty parameters combination into the HKELM model; otherwise, go to Step 5.

(8) The numerical value of the corresponding features in the test set is input into the constructed FFA-HKELM model for the forecasting of electric heating and cooling loads in the IES.

## 4. Experimental Simulation Analysis

### 4.1. Experimental Data

**Figure 7:**
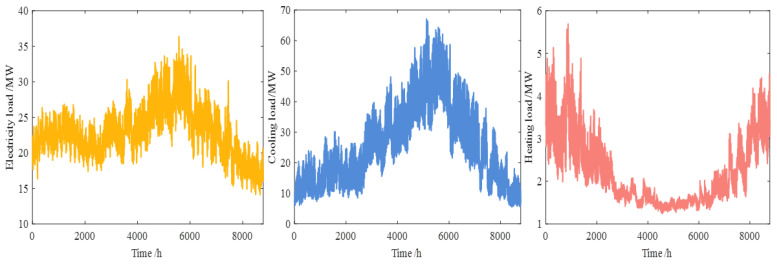
The electricity, cooling and heating loads change curves.

In the article, the measured values of the integrated energy system provided by the Tempe regional network platform of Arizona State University from 1 January to 31 December 2020 are used for the experiment [43]. The sampling period of the data is 1 h and contains a total of 8784 sample points, and the measured numerical values from 1 January to 26 December 2020 are trained to forecast electrical, heating and cooling loads from 27 December to 31 December 2020. The measured electrical, heating and cooling loads are different in dimensions, and these three loads need to be converted into a unified unit MW, where the unit relationship between different loads is 1 MW = 284 Ton = 3.4 MMBtu/h. Figure 7 shows the variations for the processed loads.

### 4.2. Model Performance Evaluation Index

So as to comprehensively and quantitatively measure the predictive performance of the model, this paper adopts three commonly used error evaluating indexes, namely mean absolute error (MAE), root mean square error (RMSE) and mean absolute percentage error (MAPE), through intuitive error analysis. The smaller the error evaluation index value, the smaller the whole error between predicted and observed values, the better the prediction performance of the model. The weighted mean absolute percentage error (WMAPE) is applied to measure the overall predictive performance of the model. The four indexes were calculated as follows:

(16) $${\mathrm{MAE}}_{r}=\frac{1}{N}\sum_{i=1}^{N}\left|{\hat{y}}_{r}(i)-y_{r}(i)\right|$$

(17) $${\mathrm{RMSE}}_{r}=\sqrt{\frac{1}{N}\sum_{i=1}^{N}{\left({\hat{y}}_{r}(i)-y_{r}(i)\right)}^{2}}$$

(18) $${\mathrm{MAPE}}_{r}=\frac{1}{N}\sum_{i=1}^{N}\left|\frac{{\hat{y}}_{r}(i)-y_{r}(i)}{y_{r}(i)}\right|\times 100\%$$

(19) $$\mathrm{WMAPE}=\sum_{r=1}^{3}(\beta_{r}{\mathrm{MAPE}}^{r})$$

where r represents the load category. $y_{r}(i)$ denotes the actual value of load type r in the IES. ${\hat{y}}_{r}(i)$ denotes the predicted value of load type r in the IES. N denotes the quantity of test samples. $\beta_{r}$ denotes the weighting coefficient of the load in category r. Due to the hot climate in the Tempe area of the United States, the demand for electricity and cooling loads is well above that for the heating load. Here, the weight coefficients are set as 0.4, 0.4 and 0.2, severally, on the basis of the importance of electric cooling and heating loads.

### 4.3. Determination of Key Parameters for the Model

In this article, HKELM is utilized for forecasting the electric cooling and heating loads in IES, while the FFA algorithm is employed to find the optimal solution of parameters in HKELM to improve prediction accuracy. Firstly, the comprehensive weight method is employed to select and determine appropriate input variables. Then, the mean square error between the fitting values from the training set and the actual values is used to construct the fitness function for optimization. The FFA algorithm is used to optimize the five parameters ω,m,n,σ,c in HKELM. By determining the optimal individual based on the maximum number of iterations, the best five parameters in HKELM are obtained. During each iteration of the FFA algorithm, the parameters within HKELM are automatically trained and optimized. The experiments are conducted on the MATLAB R2021a platform. So as to better verify the prediction performance of the proposed FFA-HKELM model, the forecasting value accuracy of this model is in comparison with the KELM, HKELM, GWO-HKELM, WOA-HKELM and SSA-HKELM models, respectively, for three different load datasets. The numerical range of the weight coefficient ω is between 0 and 1. The lower bound of the remaining parameters is [1, 0.001, 0.001, 1] and the upper bound is [20, 1000, 1000, 10]. The parameter results of HKELM optimization by various optimization algorithms are shown in Table 4, where subscripts 1, 2 and 3, respectively, represent the optimization of electrical, cooling and heating loads.

### 4.4. Analysis of Forecasting Results of Diverse Models

The FFA-HKELM model developed in this article is used to forecast the electricity, cooling and heating loads, severally. The prediction accuracy of this model is compared with the other five models through three error evaluating indexes, and the values of diverse index as illustrated in Table 5.

From Table 5, it can be found that the predicted results of the proposed FFA-HKELM model are of the highest precision, and the three evaluation indicators MAE and RMSE are all smaller than those of the other prediction models. Then, the prediction performance of each model is compared and analyzed from different angles. Compared with the result of single kernel function, the prediction precision of the model is effectively promoted by using the mixed kernel function form. The MAE of the HKELM model is reduced by 0.0342 MW, 0.0996 MW and 0.0053 MW, respectively, compared with the prediction results of the electrical, cooling and heating loads of the KELM model. RMSE is reduced by 0.052 MW, 0.1215 MW and 0.0139 MW, respectively. 

At the same time, using the optimization algorithm to optimize the key parameters of the HKELM model can significantly promote the overall predictive performance of the model. After the parameters are optimized by the FFA algorithm, the MAE of the model is reduced by 0.2772 MW, 0.2837 MW and 0.0532 MW, respectively, compared with the predicted results of the HKELM model. RMSE is reduced by 0.3335 MW, 0.3695 MW and 0.0669 MW, respectively. 

**Figure 8:**
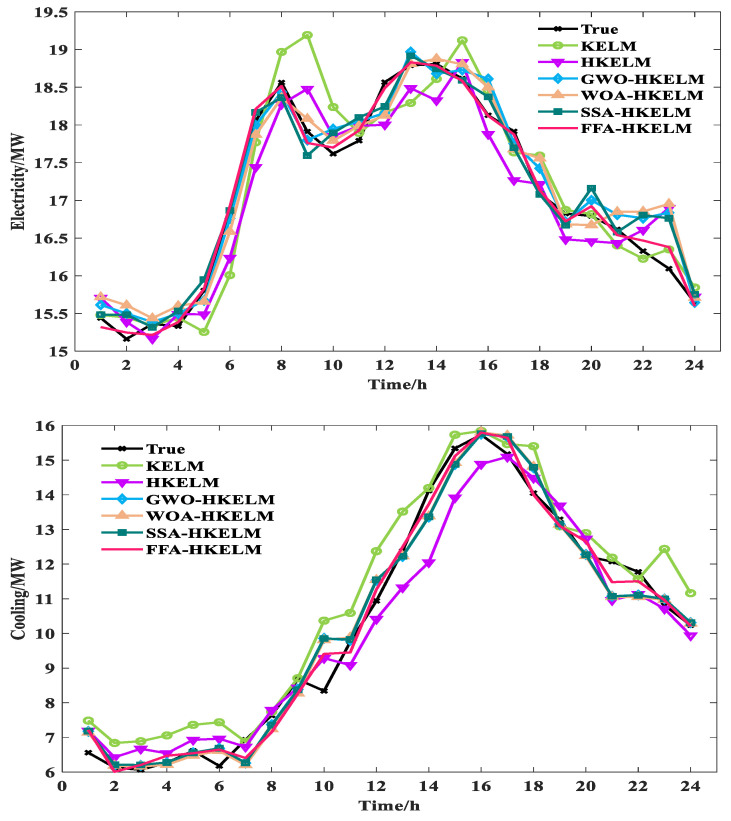

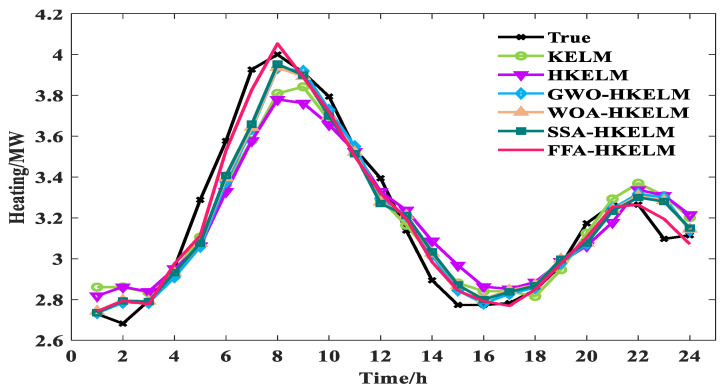
Model prediction result curves of electrical, cooling and heating loads.

Compared with the predicted results of different optimization algorithms, the MAE and RMSE of the FFA-HKELM model for electric load reductions range from 0.1147 MW to 0.1603 MW and 0.1455 MW to 0.1918 MW. For the cooling loads, the MAE and RMSE reductions in the FFA-HKELM model range from 0.1047 MW to 0.1336 MW and 0.1461 MW to 0.1707 MW. For the heating load, the MAE and RMSE reductions in the FFA-HKELM model range from 0.0247 MW to 0.0282 MW and 0.0335 MW to 0.03995 MW. It can be seen that the FFA algorithm has the best optimization-seeking effect for the HKELM, which can readily optimize the key parameters and significantly promote the predictive performance of this model. It can be found that the proposed FFA-HKELM model has the best overall prediction effect and the smallest WMAPE value. Compared with the predicted results of other models, the WMAPE reduction in FFA-HKELM model ranges from 0.9262 MW to 2.8138 MW. The predictions of these models are shown in Figure 8.

From Figure 8, it can be found that for electric, heating and cooling loads, the predicted values of the proposed model are more consistent with the real values and have the best prediction precision in comparison with the other models.

**Figure 9:**
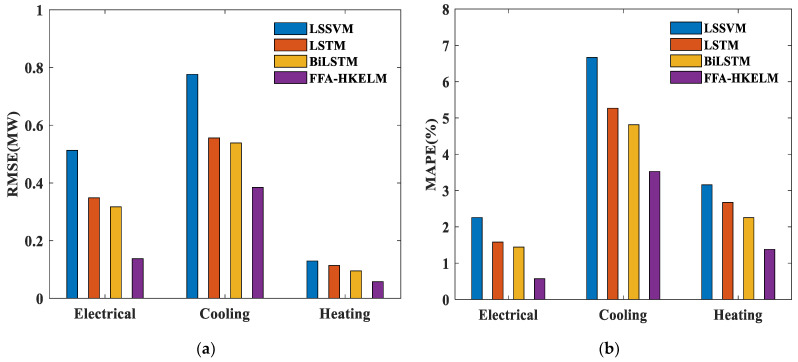
(**a**) RMSE of the FFA-HKELM model and three traditional models; (**b**) MAPE of the FFA-HKELM model and three traditional models.

Here, the FFA-HKELM model proposed in this paper is compared with the results of three traditional models, and the error indicator histograms of the results of electric, heating and cooling loads are shown in Figure 9. It can be observed that this model has the best predictive effect for electric heating and cooling loads, and obtains the smallest RMSE and MAPE value.

### 4.5. Analysis of Application Scale

The model in this article mainly uses historical load data and local meteorological data for prediction, which has a certain universality. In different application scenarios, anyone with the above data can use this model for prediction. In order to investigate the prediction performance of the model at different scales, this study also selected an industrial park and a residential area as two representative scenarios for prediction. The results of the IES multi-energy load forecasting for these three different application scales are shown in Table 6, in which the scale of the residential area is the smallest.

It can be seen from Table 6 that the model has good prediction accuracy when applied to three different scales of prediction. The model has the smallest error in IES multi-energy load forecasting for the residential area. This means that the smaller the scale of the application of the method, the better its prediction performance and exhibits higher accuracy.

## 5. Conclusions

This article presents a multivariate load forecasting method that employs the FFA-HKELM model to address the challenges of predicting electric, cooling and heating loads in an IES. It integrates the impact of various meteorological factors, enhancing the accuracy and reliability of the forecasting process. The actual operating data of energy stations in the Tempe area of the USA are utilized for application analysis. Different comparative experiments fully validate its effectiveness in multivariate load forecasting. Firstly, the comprehensive weight method combined with the entropy weight method and Pearson correlation coefficient is used to make the feature selection more effective and interpretable. This method can comprehensively measure the contribution degree and importance of features, and reduce the influence of subjective judgment and uncertainty. The model selects features that have a stronger correlation and higher information content by comprehensively considering various factors. This approach enhances the accuracy and reliability of the feature selection, thereby improving the model’s overall predictive performance. The mixed kernel function of this model not only improves the local search capability, but also enhances the global search capability indirectly through the linearly weighted Ploy kernel function and RBF kernel function. At the same time, the FFA algorithm can optimize the key penalty parameters and kernel parameters, and increase the precision of multiple load prediction. Finally, for the predicted values of electric, heating and cooling loads, the MAPE of the model is 24%~46%, 45%~74%, 43%~64% of the comparison model, severally. And under the same conditions, the MAE and RMSE of the proposed FFA-HKELM model are lower than the other models.

However, due to the limitations of data acquisition, different types of characteristic elements are not fully taken into consideration. Therefore, future studies can further consider other potential influencing factors, such as electricity demand response, prices of different energy sources and local economic material conditions, and add them to the overall forecast analysis. By measuring the degree of relevance between these factors and loads, the richness of the input features can be increased to achieve further improvements in the precision of IES multi-energy load forecasting in pursuit of broader applicability. The method presented in this paper has excellent prediction accuracy for time-varying loads such as electricity, cooling and heating. In the following research, more effective prediction methods can be proposed for random loads with temporal and spatial characteristics such as electric vehicles. This article provides a new research framework for IES multi-energy load prediction. However, due to limitations in data availability and the choice of research scenarios, this study does not consider the impact of random fluctuations from electric vehicles and distributed energy resources on the performance of load forecasting. Future work will further explore methods to enhance the performance of IES multi-energy load forecasting under conditions of multiple uncertainties.

## Figures and Tables

**Table 1:** The index weight results are obtained by entropy weight method.

Variable	Mean	Standard Deviation	CV Coefficient	Weight
Electricity	23.0376	3.6509	0.1585	0.0278
Heating	25.9396	13.7207	0.5289	0.0746
Cooling	2.1844	0.7833	0.3586	0.1017
Cloud Type	1.3947	2.5109	1.8003	0.4230
Dew Point	2.0352	7.0028	3.4408	0.0165
Surface Albedo	0.1743	0.0083	0.0474	0.0740
Wind Speed	1.9919	1.1270	0.5658	0.0528
Rainfall	1.5601	0.9266	0.5940	0.0684
Wind Direction	181.7144	85.0818	0.4682	0.0401
Relative Humidity	29.7930	18.9314	0.6354	0.0740
Temperature	23.8640	10.8537	0.4548	0.0313
Pressure	966.7251	4.3042	0.0045	0.0158

**Table 2:** Pearson correlation among all factors.

Variable	Correlation Coefficient		
	Electricity	Heating	Cooling
Electricity	1.0000	0.8024	−0.5395
Heating	0.8024	1.0000	−0.7781
Cooling	−0.5395	−0.7781	1.0000
Cloud Type	−0.0376	−0.0880	0.0580
Dew Point	0.5495	0.4983	−0.4686
Surface Albedo	0.2028	0.2819	−0.4013
Wind Speed	0.1184	0.2065	−0.0718
Rainfall	0.6592	0.6950	−0.5646
Wind Direction	0.2523	0.4025	−0.4345
Relative Humidity	−0.2482	−0.5160	0.4853
Temperature	0.6881	0.9240	−0.8260
Pressure	−0.4624	−0.5912	0.6400

**Table 3:** The comprehensive weight value of each target variable.

Variable	Electricity	Heating	Cooling
Electricity	0.0278	0.0223	0.0150
Heating	0.0598	0.0746	0.0580
Cooling	0.0549	0.0791	0.1017
Cloud Type	0.0159	0.0372	0.0245
Dew Point	0.0091	0.0082	0.0077
Surface Albedo	0.0150	0.0208	0.0297
Wind Speed	0.0063	0.0109	0.0038
Rainfall	0.0451	0.0475	0.0386
Wind Direction	0.0101	0.0161	0.0174
Relative Humidity	0.0184	0.0382	0.0359
Temperature	0.0215	0.0289	0.0258
Pressure	0.0073	0.0093	0.0101

**Table 4:** HKELM parameter optimization results.

Algorithm	C	σ	m	n	ω
$GWO_{1}$	3.3226	961.9539	971.8942	3.0092	0.3814
$WOA_{1}$	8.1971	642.9385	4.2899	2.9944	0.3181
$SSA_{1}$	8.5779	626.5937	912.1782	3.2294	0.4714
$FFA_{1}$	13.4348	265.2350	397.8229	3.7268	0.4710
$GWO_{2}$	4.3147	163.8736	175.9829	1.2052	0.1547
$WOA_{2}$	6.1788	47.6120	237.0933	1.3147	0.3590
$SSA_{2}$	13.9093	390.4754	211.7942	1.2781	0.4720
$FFA_{2}$	10.5028	399.4165	90.1655	2.3721	0.0619
$GWO_{3}$	2.1512	584.5478	48.4408	1.1163	0.2681
$WOA_{3}$	3.6612	571.3285	421.9943	1.6145	0.7435
$SSA_{3}$	8.0862	311.5170	550.3593	1.8364	0.9283
$FFA_{3}$	2.9508	639.0909	258.3997	3.1924	0.2698

**Table 5:** Prediction error results of MAE, RMSE and WMAPE.

Algorithm	Electrical		Cooling		Heating		Total
	MAE	RMSE	MAE	RMSE	MAE	RMSE	WMAPE
KELM	0.4073	0.5233	0.6936	0.8758	0.1028	0.1386	4.7288
HKELM	0.3731	0.4713	0.5940	0.7543	0.0975	0.1247	4.0767
GWO-HKELM	0.2562	0.3296	0.4439	0.5555	0.0725	0.0973	3.0884
WOA-HKELM	0.2440	0.3226	0.4194	0.5336	0.0714	0.0936	2.9518
SSA-HKELM	0.2106	0.2833	0.4150	0.5309	0.0690	0.0913	2.8412
FFA-HKELM	0.0959	0.1378	0.3103	0.3848	0.0443	0.0578	1.9150

**Table 6:** Prediction error results of different scales.

Scale	Indicator	Electricity	Cooling	Heating
Industrial Park	MAE	0.3639	0.5478	0.1227
	RMSE	0.4618	0.6738	0.1581
	MAPE	2.1329	6.1554	3.9204
University Campus	MAE	0.0959	0.3103	0.0443
	RMSE	0.1378	0.3848	0.0578
	MAPE	0.5714	3.5258	1.3805
Residential Area	MAE	0.0619	0.2494	0.0358
	RMSE	0.0825	0.3213	0.0472
	MAPE	0.4355	2.7960	1.1163

## Data Availability

The data presented in this study are available on request from the corresponding author.
